# Maintenance of crack with infilling polymer modified mortar for shear deficient of reinforced concrete beam

**DOI:** 10.1038/s41598-023-50519-0

**Published:** 2024-01-03

**Authors:** Rana Muhammad Waqas, Ayub Elahi, Mehmet Serkan Kırgız, N. Nagaprasad, Krishnaraj Ramaswamy

**Affiliations:** 1 Civil Engineering Department, University of Engineering and Technology, Taxila, Pakistan; 2 Northwestern University, Evanston, Chicago, IL 60208-001 USA; 3 Department of Mechanical Engineering, ULTRA College of Engineering and Technology, Madurai, 625104 Tamilnadu India; 4 Centre for Excellence-Indigenous Knowledge, Innovative Technology Transfer and Entrepreneurship, Dambi Dollo University, Dambi Dollo, Ethiopia; 5 Department of Mechanical Engineering, College of Engineering and Technology, Dambi Dollo University, Dambi Dollo, Ethiopia

**Keywords:** Energy science and technology, Engineering, Materials science, Mathematics and computing

## Abstract

Being a developing country, Pakistan needs sustainable and cost-effective strengthening/ retrofitting solution to be adopted/ practiced in the construction industry. The research reported in this paper was aimed to study the effectiveness of PMM, an indigenous product, for repairing reinforced concrete beams, resulting more effective and cost benefiting repair and strengthening for restoration of pre-cracked RC structures. This article presents the research results of experimental investigation conducted for repairing of cracks in shear deficient reinforced concrete (RC) beams with locally available novel material polymer modified mortar (PMM). A total of 6 beams; divided in three groups i.e. short beams, medium beams and deep beams with varying depths and same mix design were tested to four point loading under monotonic loading conditions until failure loads. Afterwards, these beams were repaired with PMM and cured with water for 72 h for retesting until failure. Load at first crack and at failure, crack pattern and deflections were recorded for all specimens during testing. Results from the experimental investigation indicate that load carrying capacity of the repaired beams was significantly restored in comparison to the control specimens. However, repaired specimens of medium group showed more improvement in load carrying capacity as compared with those of repaired specimens of short and deep group. The specimens of medium group restored up to 90% of their original load carrying capacity. The ductility is improved significantly for all shear critical repaired RC beams up to opening of cracks. Sudden brittle failure was observed after opening of repaired cracks. The contribution of PMM to load carrying capacity was found more significant for medium beams as compared to short and deep beams. The results of this study indicated that application of polymer modified mortar is effective technique for repairing of cracks in shear deficient RC beams.

## Introduction

Reinforced concrete (RC) is the most commonly used material for the construction of different types of structures including buildings, highways, bridges and dams. For a very long time, RC structures were viewed as practically eternal due to its strength, durability and repair free characteristics or minimum repairing action under certain unfavorable environmental conditions. This general perspective has changed prominently in the last two decades. Durability of concrete structures is adversely affected due to deterioration of concrete, inappropriate detailing of reinforcement, lack of maintenance activities, chemical attack, overloading and natural disasters; making the structures not serviceable for the intended design life. This requires considerable maintenance efforts for strength restoration of deteriorated RC structures. Deterioration of concrete structures can occur due to various reasons such as because of corrosion of reinforcement, carbonation, freeze and defrost^1–3^. A number of factors contribute towards the deterioration and spalling of concrete that include climate effects, age factor, loading conditions, fire and earthquake effects. Cracking and spalling are the most widely recognized reasons of the deterioration of RC structures. Cracks in concrete may happen in both the plastic state and solid state attributable to the internal stresses which emerge from the reaction of the constituent's materials to the outer excitation.

Deterioration of existing RC structures has caught the attention of various specialists to discover distinctive materials and methods to fortify and retrofit the damaged structures. Rehabilitation and enhancement in the strength of existing structures has now been developed as one of the significant exercises everywhere all around the world. Design codes of building are always being reformed as high loads on structures require increased strength of structural members. Due to the low tensile strength of concrete, American concrete institute (ACI) states in (ACI-224R-17) that cracking in concrete is unavoidable which not only leads to the exposure of reinforcement to adverse climate effects but also destroys aesthetics of the structure. This can consequently lead to distress and reduce the durability and serviceability of structure of the concrete structure^4,5^. Therefore, in order to achieve the desired service life and serviceability, repairing of these cracks become even more essential. Keeping in mind the environmental and economic point of view, these rehabilitation procedures not only prolongs the service life of current structures, but also guarantees the safety and serviceability of specific components being repaired. Understanding the reasons of cracks is important for determination of an appropriate repairing strategy. Conventional repairing techniques like concrete or steel jacketing though provide the required strength and stiffness to defective RC structural members but comes with drawbacks of increased construction time, labor intensive and costly reconstruction activities. The method of shotcrete is also a widespread repair technique where additional reinforcement and concrete layer around the existing section are provided^6,7^. Among the various deficiencies encountered while employing these conventional repairing techniques, uncertainty of the bond between the surfaces of new and existing concrete is the major disadvantage.

Steel cage has developed as an alternative to complete jacketing to avoid the drawbacks of jacketing^8–10^. The space between the existing concrete and cage is normally filled with the non-shrink grouts. Resistance against corrosion may be ensured by providing a cover of grout concrete or shotcrete. Flexure strength and shear strength can also be enhanced by the use of steel plate adhesion^11^. But there is a need of complete understanding of both short and long term performance of adhesive used for steel plate bonding. Retrofitting of existing structures can also be done by the use of composite materials^12^. There are various composite materials used for the strengthening of existing RC members but the use of Fiber Reinforced Polymers (FRP) has grown widely due to its great advantages over the other composite materials^8–10,26,27^.

Compatibility of the existing substrate and the repair materials should be considered and detailed examination of these should be conducted in advance in order to compromise the durability of repair and avoid various failure mechanism. All these issues related to choice of materials are suntil subject of research^13^. Emberson and Mays^14^ carried out research on RC beams subjected to static and cyclic loading to determine the impact of mechanical and physical properties of repair materials. The authors tested nine distinctive repair materials including epoxy, cementitious mortar and PMMs. The outcomes of the investigation indicated that properties of repair materials should be as close as possible to the substrate concrete material. The modulus of elasticity of both materials should not differ by more than ± 10 MPa and repair material should have higher tensile strength than the substrate concrete material.

Nounu and Chaudhary^15^ carried out experimental study to compare ordinary Portland cement repair with free-flowing micro-concrete repair. The results of experimental study indicated that the performance of micro-concrete repair is far better than ordinary Portland cement repair. Hassan^16^ carried out an experimental study to test the compatibility of different types of mortar repairs to concrete. Three types of mortar were studied including cementitious mortar, polymer mortar and PMM. It was concluded from the study that difference in modulus of elasticity of repair material and concrete lowers the load carrying capacity of combined system. Cementitious mortar is less effective as repair material due to its high shrinkage. While PMM is the most appropriate repair material due to lower shrinkage and compatibility of modulus of elasticity with substrate concrete. Mangat and O’Flaherty^17^ used seven different ordinary and polymer modified cementitious mortars to repair two existing bridges. The results indicated that repair material having greater stiffness performed well than other materials. However in any case the difference in elastic modulus of repair material and substrate concrete was not more than ± 10 MPa as suggested by Emberson and Mays^14^. Besides, the most performing materials were described by the most reduced shrinkage properties.

Rı'o et al.^18^ conducted study on flexure deficient beams after simulating corrosion at mid span and patch repair with three different types of mortar including cement based, epoxy adhesive binder and PMM. They described that load carrying capacity of the repaired beams was slightly lower than control beams but higher than the damaged beams. They also concluded that the properties of repair material should be close enough to substrate concrete. Park and Yang^19^ conducted study on eight RC beams repaired with ordinary Portland cement and PMM. The repair material was applied in the tension region of all beams. The variables investigated in this study were the reinforcement ratio and length of repair patch. The results given by Portland cement repair were satisfactory but the PMM repairs gave the best results in terms of load carrying capacity and ductility. Sharaf et al.^20^ made use of a capsulated healing agent implanted in mortar matrix in order to obtain self-healing properties of concrete mix, for repair of concrete structures. The idea was that, when structure is subjected to loading and cracks, the capsule will break up leading to the release of healing agents. The process of crack filling and healing was observed by using a combination of visual observation and tomography. Authors indicated that application of the proposed methodology led to a gain of 50% of the original strength and stiffness. It was also found that use of the proposed methodology could lead to increased initial costs, but on the other hand, maintenance costs could be reduced and life of structures could be increased. Shannag and Al-Ateek^21^ carried out experimental investigation on 30 RC beams. The beams were repaired with five different types of materials applied only in the tension region. The materials used for repairs were ordinary Portland cement and four types of fiber reinforced cementitious materials. Once repaired, the beams were retested as they were or after accelerated corrosion. The results from this study indicated that the beams which were repaired with ordinary Portland cement gave the worst results among all repair materials before and after inducing corrosion.

Ahmad et al.^4^ made use of PMM to control cracking in RC beams. The authors proposed a novel retrofitting technique for pre-cracked RC members, for a developing country like Pakistan with limited resources. PMM is claimed to be a reliable and cost effective material for the said purpose. For the experimental program, the authors constructed six full scale RC beams, all with same materials and binder ratios. All these were put to test load conditions in order to develop cracks up to 1 mm limiting value. Once this was achieved, repair process was started with the aid of PMM. Curing was performed for a period of 3 days, after which all beams were again tested and loaded until failure. Results indicated significant improvement in load carrying abilities of the constructed beam samples, which clearly demonstrated that PMM could efficiently be used as a repair/retrofitting material for damaged concrete structures. Kim et al.^22^ tested RC beams with fiber reinforced cementitious mortar applied at the intrados of RC beams. The beams were designed with and without shear reinforcement. After applying the repair, the beams that were intended to fail in shear reached the same ultimate load and deflection of beams having shear reinforcement. When the thickness of repair was doubled, the capacity of beams without shear reinforcement reduced to half due to interface failure. But the beams that were designed to fail in flexure could restore the load carrying capacity of original beam with both types of repair thickness.

There is enough research available on the use of PMM to enhance the properties of non-damaged and non-repaired beams. But the use of this material for strengthening/retrofitting of damaged RC beam are not well reported; even no adequate research literature is available on its use as strengthening/retrofitting material for damaged RC beams. Being a developing country, Pakistan also needs sustainable and cost-effective repairing solution practices for RC structural elements to be adopted in the construction industry. Therefore, an experimental investigation has been planned to evaluate the behavior of RC beams retrofitted with PMM which has not been a focus of application until now.

Conventional upgrading techniques often lead to heavy demolition, lengthy construction time, reconstruction, and relocation of inhabitant with all the related costs (direct and indirect). Huge indirect costs, the environmentally hostile approach and the hassle associated with conventional techniques are some of the major reasons that discourage the relevant stake holders of buildings from their commitment regarding strengthening/retrofitting. Further, the usage of PMM as strengthening/retrofitting material for RC members is not well documented; no sufficient research data is available on use of PPM as strengthening/retrofitting material except some successful case histories of repair. Such current case histories and results on some of its other properties encouraged the authors to investigate performance behaviour of the RC beams using PMM. Being a developing country, Pakistan also needs sustainable and cost-effective strengthening/ retrofitting solution to be adopted/ practiced in the construction industry. The research reported in this paper was aimed to study the effectiveness of PMM, an indigenous product, for repairing reinforced concrete beams, resulting more effective and cost benefiting repair and strengthening for restoration of pre-cracked RC structures. The proposed technique includes application of PMM in cracked beams, before failure, to enhance the load carrying capacity. It was observed that even failed beams restored the capacity to some extent after their retrofitting with PMM. The study will enhance the data base towards sustainable solution to the fully/partially deteriorated concrete structures. The study will help in identifying the increase in load carrying capacity of shear deficient RC beams which are retrofitted with PMM and subjected to various loading conditions.

## Experimental program

The experimental program was carried out to better explain the effectiveness of PMM for repairing of shear critical RC beams. Total reinforced concrete beams 6 were cast with different depths 3 such as 300 mm (short beams), 375 mm (medium beams) and 450 mm (deep beams) to evaluate the effectiveness of PMM for retrofitting of beams having different cross sections. The following sections would describe the materials, mix proportions, details of beam specimens, experimental setup, and retrofitting scheme used.

### Materials

#### Materials for concrete

Ordinary Portland cement (OPC) conforming to American society of testing and materials (ASTM) Type 1 as per rule of ASTM C150/C150M standard was selected for concrete production^23^. Coarse aggregate and fine aggregate, which were respectively taken from the Margalla and the Lawrencepur sources in Pakistan, were used for concrete constituent. The maximum size of coarse aggregate used was 20 mm. Recycled-steel, known as reinforcement material for concrete in beam specimen, was used for preparation of beams. The recycled steel bars—D16 and D20 and D10 used have yield strength 445 MPa and 300 MPa, respectively.

#### Materials for repair

PMM was used for the strengthening/retrofitting of beam specimens. PMM was injected into the cracks of beams as per manufacturer recommendations. Before injection of PMM, deteriorated concrete and dust were removed to ensure proper bond between concrete substrate and PMM. Properties of PMM are described in Tables 1 and 2.

**Table 1:** Properties of fresh mortar (ordinary mortar and polymer modified mortar).

Sr. No.	Properties	Polymer modified mortar	Ordinary mortar
1	Unit weight (kg/l)	1.9	2.0
2	Air content (%)	8.2	6.1
3	Water retention(%)	96.6	70

**Table 2:** Properties of hardened mortar (ordinary mortar and polymer modified mortar).

Sr. No.	Properties	Polymer modified mortar	Ordinary mortar
1	Total pore volume (× 10^−2^ cm^3^/g)	10.3366	11.2531
2	28 days compressive strength (kgf/cm^2^)	520	234
3	Max. deflection (× 10^–1^ mm)	1.0	0.42
4	Max. extreme tensile fiber strain (× 10^–6)^	1231	385
5	Max. tensile strain (× 10^–6^)	380	82
6	Flexural modulus of elasticity (× 10^4^ kgf/cm^2^)	6.31	7.36
7	Tensile modulus of elasticity (× 10^5^ kgf/cm^2^)	2.27	2.63
8	Crack coefficient (× 10^−2^ cm^2^/kg)	0.020	0.037
9	Adhesion in tension (kgf/cm^2^)	22	5
10	Water absorption (%)	9.3	12.2
11	Water permeation (g)	6	66
12	Freeze–Thaw durability factor	72	10
13	91-days carbonation depth (mm)	10	21

PMM is a revolutionary, multifunctional, high-tech, high performance, sustainable, durability improving constructional material for twenty-first century and belongs to the category of “Concrete—Polymer Composites”. PMM is extremely vital for durable construction and is a proportionate mixture of cement, sand, various advanced polymers, super plasticizers, shrinkage reducing and water repelling agents. Only water is required to be added to PMM before use and hence it is ready to use construction material. A monolithic effective plastic lining system of polymer films is automatically developed in PMM upon addition of water which is responsible for its improved behavior.

**Figure 1:**
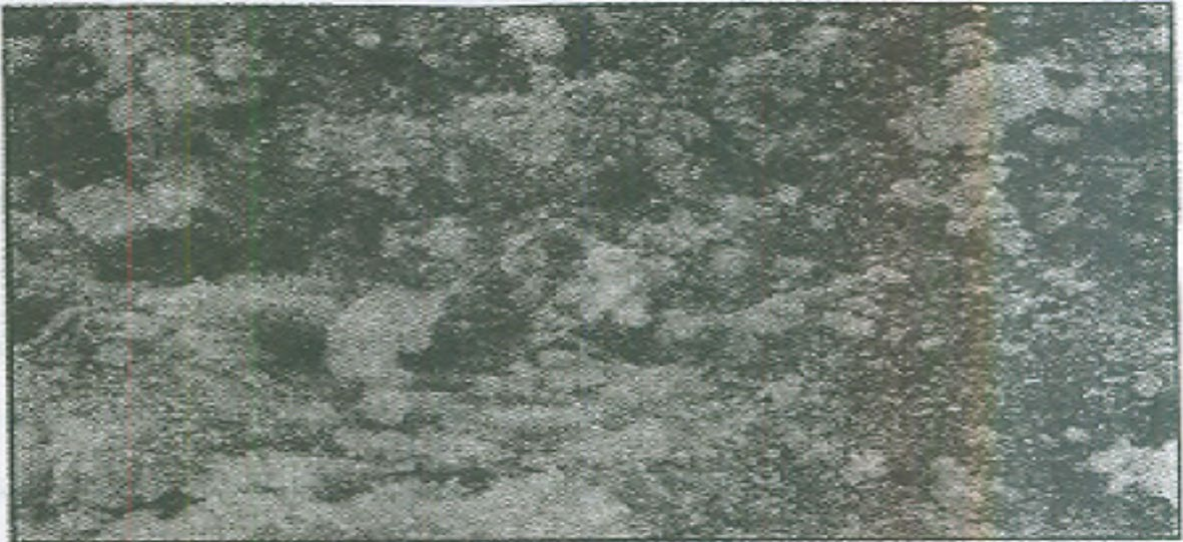
Electron micrographs of unmodified mortar.

**Figure 2:**
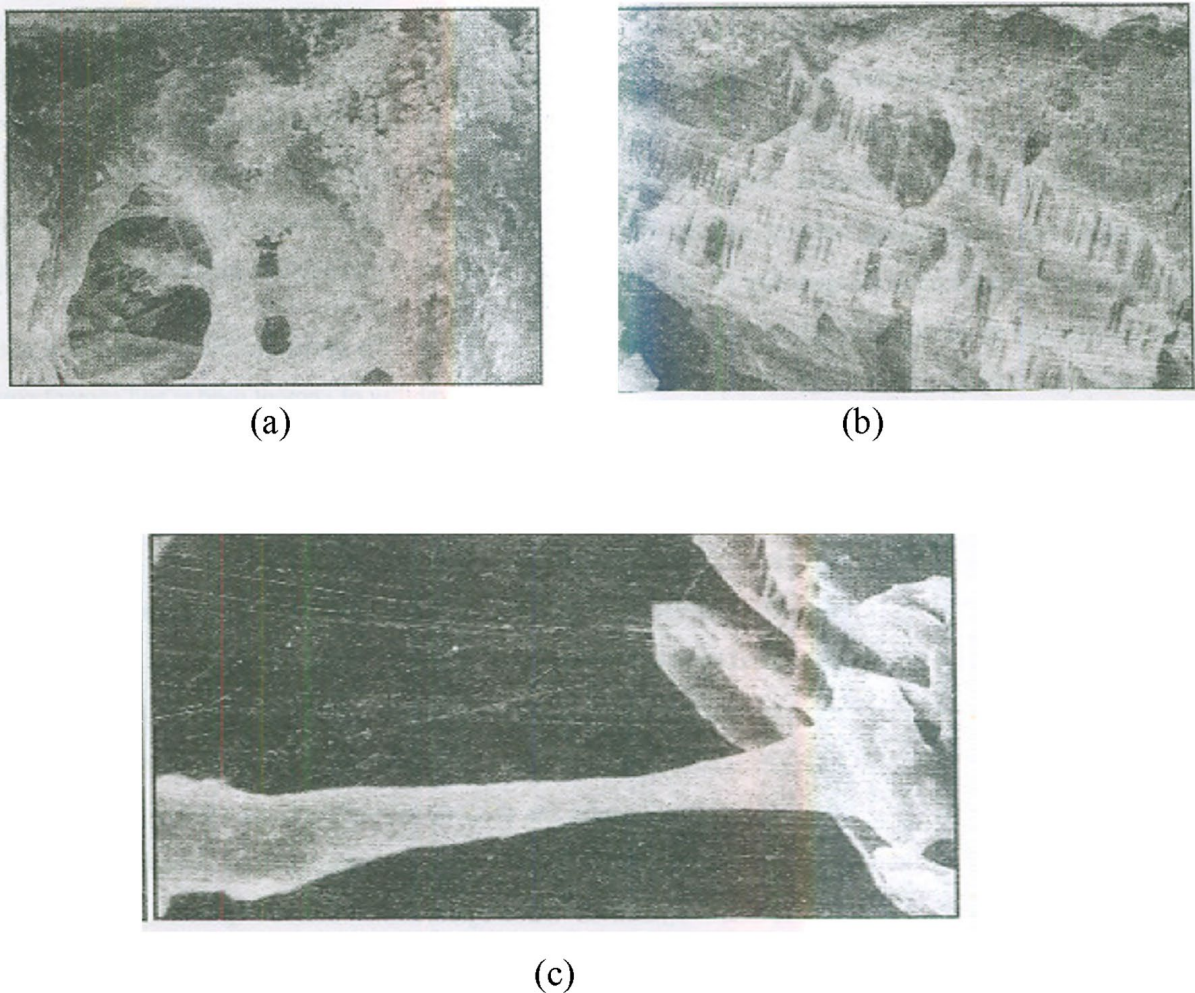
(**a**–**c**) Electron micrographs of PMM.

Figure 1 shows that in the absence of polymer films, unmodified mortar is of poor quality. Figure 2 shows that due to presence of monolithic network of polymer films in PMM, its mechanical properties and durability are greatly improved in comparison to the un-modified mortar.

### Mix proportions

The concrete mix ratio was kept as 1:2.4 (cement: sand: aggregate) by performing trials to attain a concrete having targeted compressive strength equal to 30 MPa at 28 days. The water cement ratio (w/c) was used as 0.42. Two concrete cylinders were casted to calculate the strength of concrete after 28 days^24^. These cylinders were kept in hessian cloth along with beams for curing purposes.

Cylinders were taken out of hessian cloth after 28 days and kept in air for drying purposes. Then these cylinders were tested and an average compressive strength of 31 MPa was recorded after 28 days. Slump cone test was carried out to find out the workability of concrete and a slump value of 80 mm was achieved^25^.

### Detail of beam specimens

The research reported in this paper was aimed to study the effectiveness of PMM, an indigenous product, for repairing shear deficient reinforced concrete beams, resulting more effective and cost benefiting repair and strengthening for restoration of pre-cracked shear deficient RC structures. All the specimens were designed in this study to fail in shear, so they were designed with shear deficient reinforcement.

Total 6 full-scale RC beams were constructed. All specimens were designed as shear critical by ignoring the shear reinforcement. The beams were divided in three groups depending upon their depths designated as short beams for beams with depth of 300 mm, medium beams for beams with depth of 375 mm and deep beams for beams having depth of 450 mm. Each group consists of two beams with identical depths. Total span and width were kept same as 2750 mm and 225 mm for all specimens. The beam grouping, dimensions and reinforcement details are shown in Table 3.

**Table 3:** Detail of beam specimens.

Beam group	Beam ID	Average concrete strength (MPa)	Clear span (mm)	Depth of beam (mm)	a/d
Short beams	S-1	31	2400	300	2.70
	S-2	31	2400	300	2.70
Medium beams	M-1	31	2400	375	2.70
	M-2	31	2400	375	2.70
Deep beams	D-1	31	2480	450	2.70
	D-2	31	2480	450	2.70

**Figure 3:**
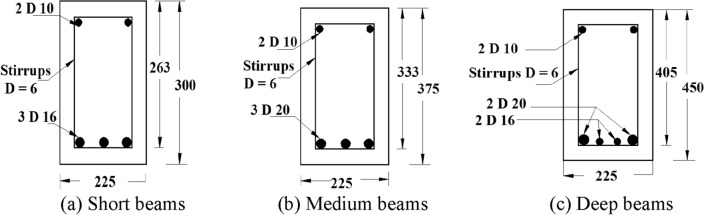
Transverse section of beams.

**Figure 4:**
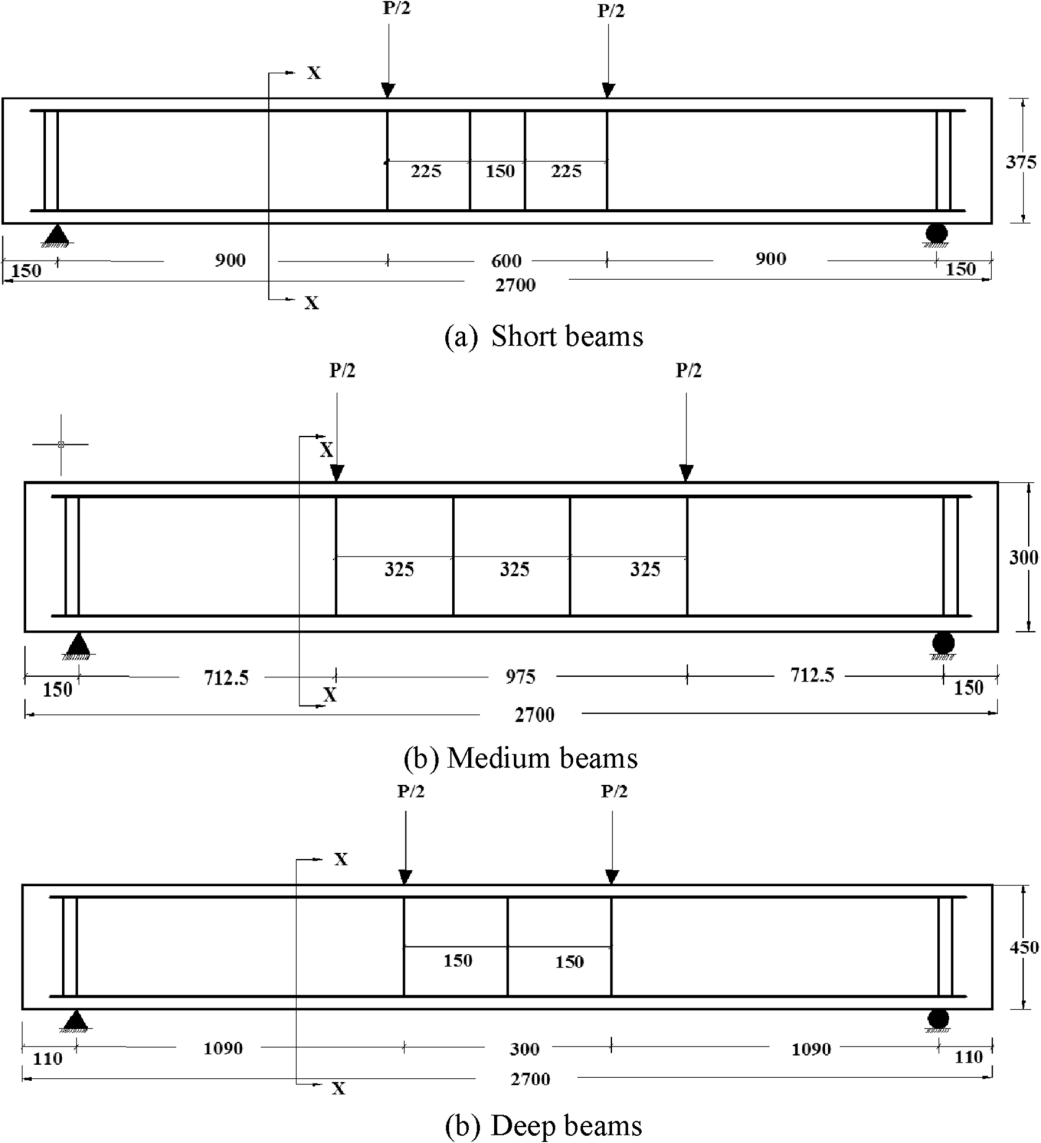
Longitudinal section of beams.

Reinforcement ratio and a/d were kept constant in all specimens. All beams were tested until failure load and the results of the control model were compared with repaired beam models. The control beams’ group is suffixed with letter ‘C’ in the beam group names. The specimens of short beams were named as SDS-1 and SDS-2, medium beams as SDM-1 and SDM-2, deep beams as SDD-1 and SDD-2. The cross sections, reinforcement and loading details of all beams are shown in Figs. 3 and 4.

### Preparation of test specimens

All beam specimens were casted in steel forms. They were moved out of forms after one day of casting and covered in wet cloth for curing purpose. Curing of specimens was continued for 28 days. After 28 days, specimens were taken out of wet cloth and kept in air for one day for drying.

### Test setup and instrumentation

**Figure 5:**
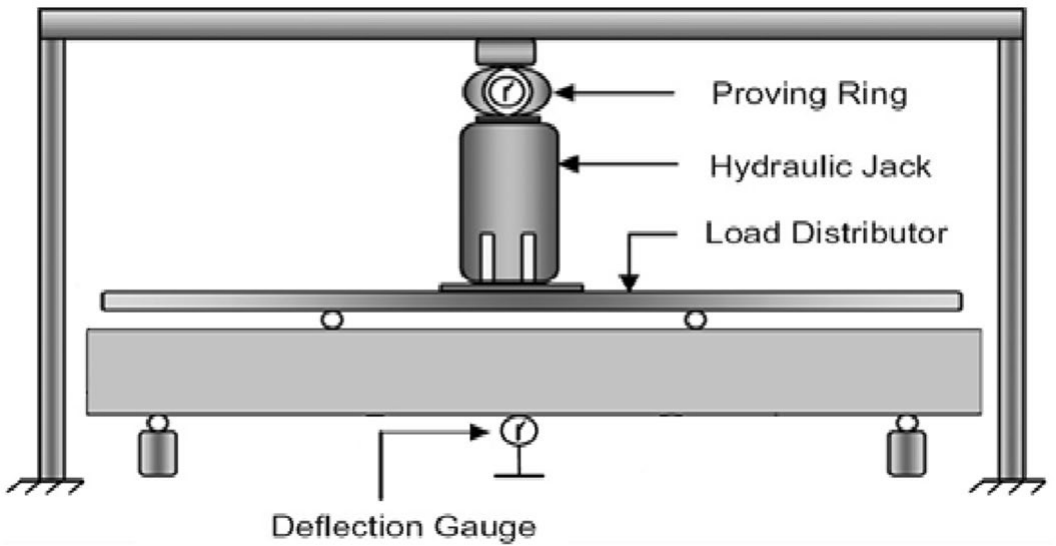
Detailed illustration of test setup.

All beams were tested to four-point monotonic loading under simply supported conditions as shown in Fig. 5. Load was applied in intervals at rate of 5 kN/min using a hydraulic jack having a capacity of 1000 kN. Load under the hydraulic jack was used as load distributor to generate the two-point loads. Deflection gauges were employed to determine the deflection of specimens. The deflection was recorded very carefully after the application of each load increment. To avoid the chance of error, two dial gauges were employed to measure the mid-span deflections. Dial Gauges are basic instruments that are used to measure accurately very small and diminutive liner distances. These instruments are reliable and are also used by the different researchers around the globe to measure the linear deflections in different structural elements as evident from number of published data.

### Retrofitting/strengthening configurations

The beam specimens designed in this experimental program were shear critical i.e. beams deficient in shear strength by ignoring the shear reinforcement. The aim of retrofitting/ strengthening was to improve the strength of specimens in shear.

**Figure 6:**
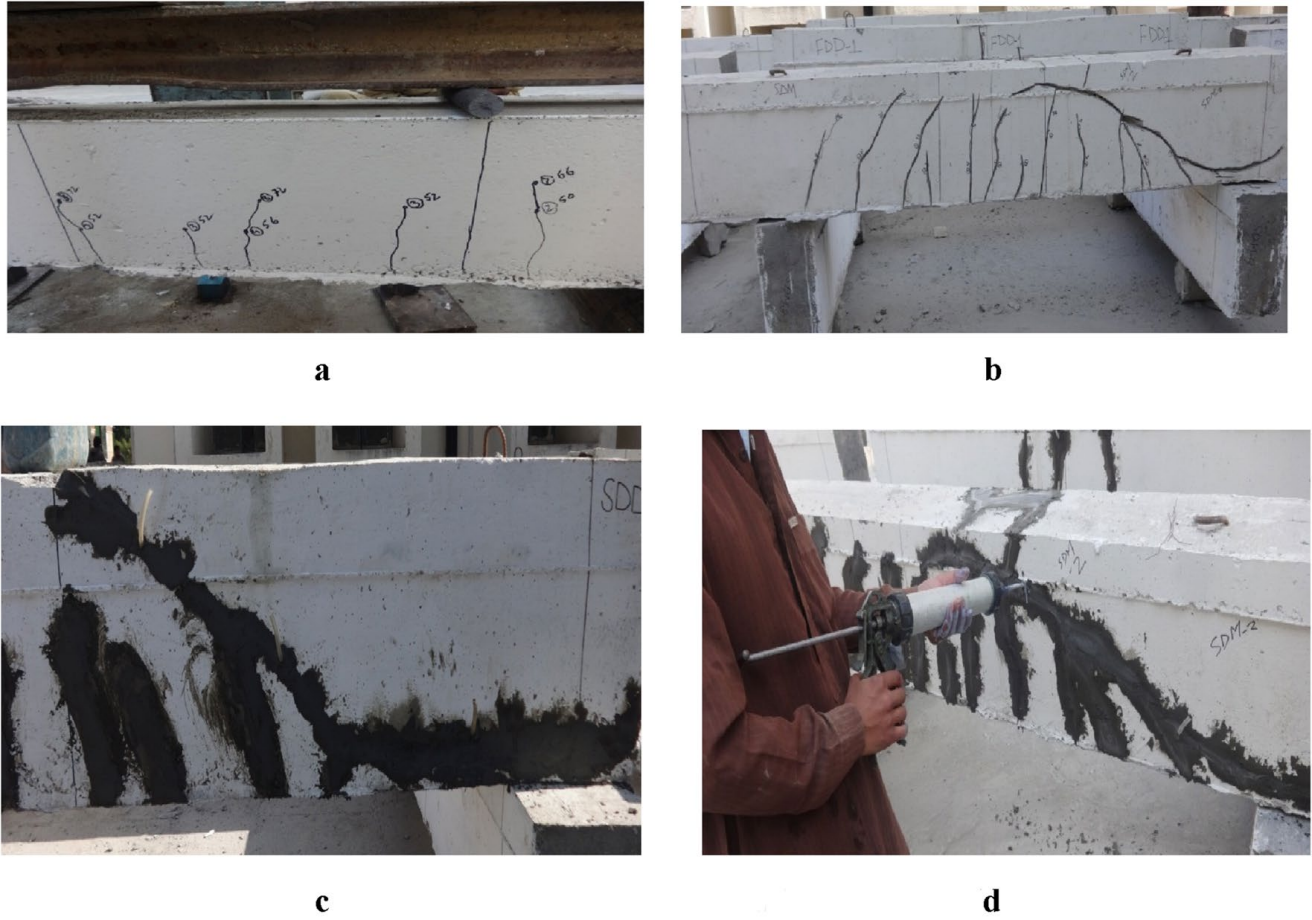
(**a**) Crack marking with respective load. (**b**) cleaning cracks and preparing for fill. (**c**) Sealing of cracks with PMM paste. (**d**) Injection of PMM into cracks.

This program was intended to repair cracks developed after initial testing in beams. Sudden brittle failure was observed in all beams due to wide diagonal tensile cracks. No diagonal shear crack was observed before failure. Only hairline vertical flexure cracks were noted. After initial testing all beams were repaired with PMM. Before applying PMM to Beams, all areas of damaged and cracked concrete were identified, delineated and clearly marked out prior to commencement of works. All traces of existing coatings, sand or cement rendering were removed from the damaged areas in order to properly assess the damage area as shown in Fig. 6b.

Water was then added to PMM to prepare a paste of enough consistency which was used to seal all the cracks as shown in Fig. 6c. After sealing the cracks, flow-able mix was prepared to inject the mixture into cracks through nozzles under pressure using a pressure gun as shown in Fig. 6d. Procedure of repairing of cracks with PMM is illustrated in Fig. 6 (a-d). After repairing process, these specimens were retested until failure and all data were recorded for each specimen including first crack load, failure loads, failure modes and mid span deflection.

## Results and discussion

In this section, the results from experimental study are discussed. The comparison between controlled and PMM repaired beams results were made through load deflection plots, loads at first crack, loads at failure.

### Effect of repairing material on loads at first crack

**Figure 7:**
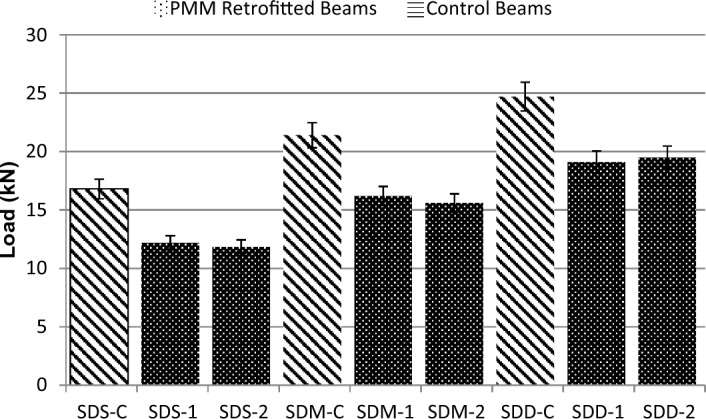
Comparison of loads at first crack of controlled and repaired beams.

Load required to generate the first crack was noted in each specimen. Figure 7 shows the comparison between loads at first crack of control and PMM repaired specimen. It can be seen from the Fig. 7 that deep beams tend to crack and eventually fail at relatively larger loads in comparison to medium and short beams. Also, loading values at first crack after repair in all beams were comparatively lower than controlled beams; concluding that the use of PMM as strengthening material could not restore the original strength of specimens.

On a detailed note, the percentage difference of first crack loads were higher for medium beams as compared to deep and short ones. For short beams, these were found to be 49.81% respectively lower than controlled beams. For medium beams these were 62.40% lower as compared to controlled beams. As far as deep beams are concerned, the percentage difference was found to be 59.64% and 51.21% respectively lower, in comparison to controlled beams.

### Effect of repairing material on failure modes and ultimate loads

**Figure 8:**
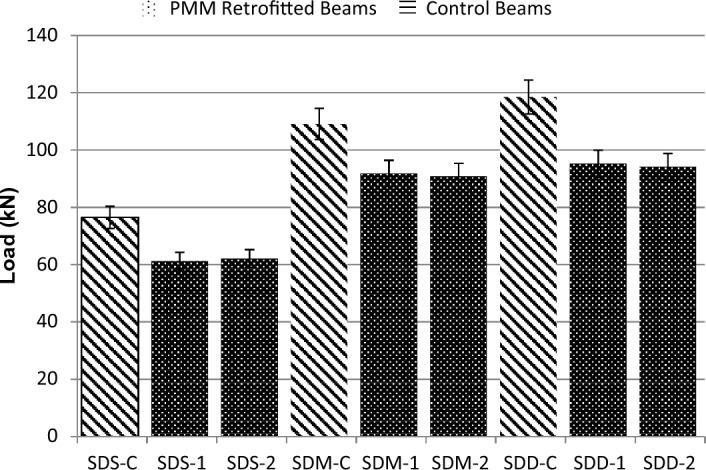
Comparison of failure loads for control and repaired beams.

The failure loads obtained from the experimental results along with relative strength as compared to control specimens are shown in Table 4 and Fig. 8 for short, medium and deep beams.

**Table 4:** Ultimate load of beams with relative strength.

Beam ID	Ultimate load (kN)	Relative strength (as compared to respective control specimen)
SDS-C	76.5	–
SDS-1	61.25	0.80
SDS-2	62.1	0.82
SDM-C	109.1	–
SDM-1	96.8	0.88
SDM -2	98.8	0.90
SDD-C	118.1	–
SDD-1	95.2	0.80
SDD-2	94.1	0.79

All control beams tested before the application of PMM were failed in shear with no sign of ductility due to absence of stirrups. At failure, wide shear diagonal cracks were observed in all specimens. Vertical flexure cracks were developed in the region where stresses exceeded the modulus of rupture.

All specimens, after repairing with PMM showed similar behavior at failure. All specimens were failed due to the cracks over the shear zone. The possible reason of this failure mode in all specimens of was the opening of repaired shear diagonal cracks which suddenly increased the stresses that lead to the sudden widening of repaired cracks. All specimens when reached about 80–90% ultimate load of control specimens, opening of the repaired shear crack occurred leading to failure of specimens. In short beams group, specimens, SDS-1 and SDS-2, failure occurred due to opening of shear cracks at applied load of 61.5 kN and 62.5 kN; both specimens restored about 80% strength of control beam SDS-C. Specimens of medium group beams, SDM-1 and SDM-2 failed at applied load of 98.8 kN and 96.8 kN due to reopening of shear crack. In both specimens, strength restored after PMM application was 90% and 88% of control beam SDM-C.

Specimens of deep group beams, SDD-1 and SDD-2 failed due to widening of shear crack followed by the crushing of concrete in compression zone at applied load of 95.2 kN and 94.1 kN. Both specimens regain about 80% strength of control beam SDD-C. It was observed from the experimental results that cross sections of beams had a very less effect on the performance of PMM for repairing of cracks in shear critical beams. The maximum load was restored by the medium group beams as compared to small and deep group beams.

### Load deflection behavior

**Figure 9:**
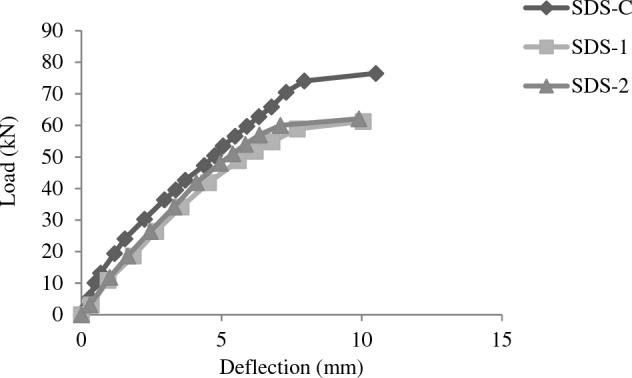
Load deflection plots for beams of SDS group.

**Figure 10:**
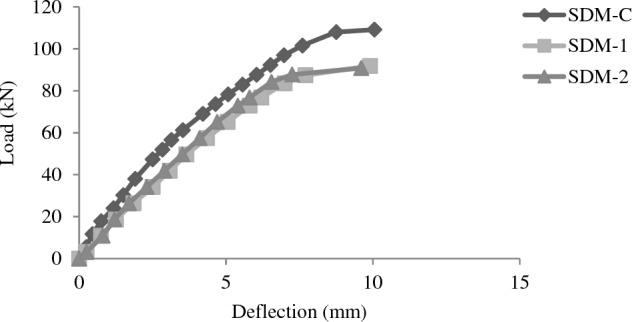
Load deflection plots for beams of SDM group.

**Figure 11:**
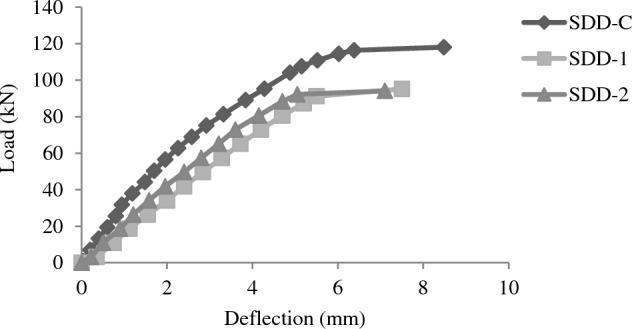
Load deflection plots for beams of SDD group.

Deflection gauges were employed to measure the mid span deflection for all test beams, deflection was taken at the same points and after each load increment for all the test beams. Comparisons were consequently made for each load increment for all cases of short, medium and deep beams. Comparison plots are shown in following Figures 9, 10, 11.

For the case of short beams (SDS group), Initial load/deflection curve is much flatter in control beams. But as controlled beams move towards failure, enhanced deflection is observed until failure.

Load deflection plot of PMM repaired beams is less stiff as compared to control beams at the initial stages. But PMM repaired beams show a much smoother curve with smaller deflection at failure loads indicating improved stiffness of beams as compared to controlled beams at failure point. Short beams showed sudden increase in deflection after about 75% of ultimate load. Maximum ultimate load restored is 82% of that of control beam.

As far as medium beams (SDM) group are concerned, smooth linear load deflection curve is observed in repaired beams until the point of opening of cracks. Both beams show 13% less deflection at failure with maximum ultimate load restored is 82% of that of control beam.

For the case of deep beams (SDD) group, linear load deflection curve is observed in repaired beams. Linear load deflection trend is observed up to 70% of failure load. But sudden increase in deflection after about 75% of ultimate load is observed deep beams. The deflection at failure for deep group beams was lower for controlled beams. Both beams show 33% less deflection at failure compared to control beams indicating improved stiffness of beams as compared to control beams.

## Conclusion

This study has investigated the role of a novel material PMM for repairing of shear critical RC beams. The beams of different cross sections were selected for this purpose. Important conclusions drawn from this study are given below:

- Repairing technique using PMM application can be used as an effective means of repairing the cracks of shear critical RC beams.
- Application of PMM helps in restoring about 90% of the load carrying capacity of shear critical RC beams.
- The ductility is improved significantly for all shear critical repaired RC beams up to opening of cracks. Sudden brittle failure was observed after opening of repaired cracks.
- Contribution of PMM to load carrying capacity was found more significant for medium beams as compared to short and deep beams.
- All the beams were failed due to opening of repaired cracks which strongly suggests that application of PMM is effective in the restoration of strength of cracked concrete structures.

The results of this study are only valid for shear deficient beams. They may not be applicable to flexure deficient beams. The effectiveness of PMM may be studied for flexure deficient beams and other RCC structural elements i.e., columns, slabs, and walls. Furthermore, a comprehensive investigation may be carried out to compare the effectiveness of PMM with carbon fiber reinforced polymer (CFRP) laminates.

## Data Availability

The datasets used and analyzed during the current study are available from the corresponding author on request.

## References

[1] Song H-W, Kwon S-J, Byun K-J (2006). Park C-K predicting carbonation in early-aged cracked concrete. Cem. Concr. Res..

[2] Sun W, Mu R, Luo X (2002). Miao C Effect of chloride salt, freeze–thaw cycling and externally applied load on the performance of the concrete. Cem. Concr. Res..

[3] Fang C, Lundgren K, Chen L (2004). Zhu C Corrosion influence on bond in reinforced concrete. Cem. Concr. Res..

[4] Ahmad S, Elahi A, Barbhuiya S (2012). Farid Y Use of polymer modified mortar in controlling cracks in reinforced concrete beams. Constr. Build. Mater..

[5] Craeye B, Geirnaert M (2011). De Schutter G Super absorbing polymers as an internal curing agent for mitigation of early-age cracking of high-performance concrete bridge decks. Constr. Build. Mater..

[6] Chapdelaine F (2000). Beaupre D Pumping fiber-reinforced wet-mix shotcrete. Am. Shotcrete Assoc. Mag..

[7] Heere R, Morgan DR, McAskill N (2002). Determination of early-age compressive strength of shotcrete. Shotcrete Mag..

[8] Georgopoulos, T. & Dritsos, S. A steel jacketing technique for strengthening RC columns. In *Proceedings of the 10th European Conference on Earthquake Engineering* 2275–2280 (1994).

[9] Dritsos, S. & Pilakoutas, K. Repair/strengthening techniques for structurally damaged RC columns. In *Proceedings of the 5th US National Conference on Earthquake Engineering* 667–676 (1994).

[10] Prasad, N. Enhancement of flexural capacity of undamaged RC column by a composite steel caging technique. M Tech Thesis, Indian Institute of Technology Kanpur, India (2005).

[11] Hashim S (1999). Adhesive bonding of thick steel adherends for marine structures. Mar. Struct..

[12] Said AM, Nehdi M (2004). Use of FRP for RC frames in seismic zones: Part I. Evaluation of FRP beam-column joint rehabilitation techniques. Appl. Compos. Mater..

[13] Ohno K, Ohtsu M (2010). Crack classification in concrete based on acoustic emission. Constr. Build. Mater..

[14] Emberson N, Mays G (1990). Significance of property mismatch in the patch repair of structural concrete Part 1: Properties of repair systems. Mag. Concr. Res..

[15] Nounu G (1999). Reinforced concrete repairs in beams. Constr. Build. Mater..

[16] Hassan K, Brooks J, Al-Alawi L (2001). Compatibility of repair mortars with concrete in a hot-dry environment. Cem. Concr. Compos..

[17] Mangat P, O'Flaherty F (2000). Influence of elastic modulus on stress redistribution and cracking in repair patches. Cem. Concr. Res..

[18] Rio O, Andrade C, Izquierdo D, Alonso C (2005). Behavior of patch-repaired concrete structural elements under increasing static loads to flexural failure. J. Mater. Civ. Eng..

[19] Park S, Yang D (2005). Flexural behavior of reinforced concrete beams with cementitious repair materials. Mater. Struct..

[20] Sharaf MH, Soudki KA, Van Dusen M (2006). CFRP strengthening for punching shear of interior slab–column connections. J. Compos. Constr..

[21] Shannag MJ, Al-Ateek SA (2006). Flexural behavior of strengthened concrete beams with corroding reinforcement. Constr. Build. Mater..

[22] Kim J-HJ, Lim YM, Won J-P, Park H-G, Lee K-M (2007). Shear capacity and failure behavior of DFRCC repaired RC beams at tensile region. Eng. Struct..

[23] Astm C 150. Standard specification for Portland cement. Annual book of ASTM standards 4 (2002).

[24] ASTM A. ASTM C39/C39M-18: Standard test method for compressive strength of cylindrical concrete specimens. ASTM International, West Conshohocken, PA ASTM, AI (2018) ASTM C 192 (2018).

[25] ASTM C. 143/C 143 M-12. Standard Test Method for Slump of Hydraulic-Cement Concrete, ASTM International (2012).

[26] Hawileh RA, Rasheed HA, Abdalla JA, Al-Tamimi AK (2014). Behavior of reinforced concrete beams strengthened with externally bonded hybrid fiber reinforced polymer systems. Mater. Design.

[27] Hawileh R, Al-Tamimi AK, Abdalla JA, Wehbi MH (2011). Retrofitting pre-cracked RC beams using CFRP and epoxy injections. Key Eng. Mater..

